# Investigation of Ocular Blood Flow in Males with Metabolic Syndrome

**DOI:** 10.3390/diagnostics15162021

**Published:** 2025-08-12

**Authors:** Takahiro Maruyama, Tomoaki Shiba, Tatsuhiko Kobayashi, Seiji Takagi, Yuichi Hori

**Affiliations:** 1 Department of Ophthalmology, Tokyo Shinagawa Hospital, Tokyo 140-8522, Japan; t-maruyama@med.toho-u.ac.jp; 2 Department of Ophthalmology, Toho University Graduate School of Medicine, Tokyo 143-8541, Japan; yhori@med.toho-u.ac.jp; 3 Department of Ophthalmology, International University of Health and Welfare, Narita 286-8520, Japan; 4 Department of Ophthalmology, Toho University Omori Medical Center, Tokyo 143-8541, Japan; tatsuhiko.kobayashi@med.toho-u.ac.jp (T.K.); seiji.takagi@med.toho-u.ac.jp (S.T.)

**Keywords:** laser speckle flowgraphy, metabolic syndrome, ocular blood flow, imaging, optic nerve head, choroid

## Abstract

**Objectives:** This study aims to investigate ocular blood flow in male subjects with metabolic syndrome (MetS) using laser speckle flowgraphy (LSFG). **Methods:** Subjects who underwent LSFG ocular blood flow testing during physical examinations were separated into a MetS group and a control group. The groups were propensity score-matched by age, with 138 male subjects compared in each group. The subjects’ ocular blood flow (mean blur rate, MBR) in the optic nerve head (ONH) and choroid was measured via LSFG. Pulse waveform parameters, the blowout score (BOS), blowout time (BOT), and rising rate (RR), were also measured. The ONH region was measured as a whole and as tissue and vascular regions. **Results:** The MBR-Choroid was significantly lower in the MetS group versus the control group. There was no significant difference in the MBR in the ONH. Compared to the control group, the RR values in the MetS group were significantly lower in all regions. The whole tissue region and vascular region BOS values were significantly higher in the MetS group. A single-regression analysis revealed that among the evaluated parameters, only the number of MetS components was significantly negatively correlated with the MBR-Choroid. A multiple regression analysis identified HbA1c as a factor contributing independently to the MBR-Choroid among the MetS-related factors. **Conclusions:** This investigation of adult males clarified that in the early stage of MetS, the MBR in the choroid area decreases in parallel with the accumulation of MetS components. The MetS component with the strongest influence on the MBR-Choroid was HbA1c.

## 1. Introduction

Metabolic syndrome (MetS) involves a group of risk factors for atherosclerosis that include obesity, glucose intolerance, dyslipidemia, and hypertension. The prevalence of MetS has been increasing yearly due to the prevalence of unhealthy diets and a lack of exercise, and MetS has become a social problem due to the increased risk of atherosclerotic disease and death in several populations [1]. Since MetS has a complex etiology and ultimately leads to irreversible cardiovascular dysfunction [2,3], early detection and the prevention of its progression are particularly important during the stage in which vascular dysfunction is reversible. In ophthalmology, it is recognized that MetS is associated with several ophthalmic diseases, such as retinal vein occlusion development and the progression of diabetic retinopathy [4,5].

Laser speckle flowgraphy (LSFG) is a noninvasive technique that can be used to reproducibly quantify ocular blood flow [6,7,8]. The measured ocular blood flow value is displayed as the mean blur rate (MBR). The MBR has been reported to be influenced by background and systemic conditions such as age-related changes and sex differences [9,10,11]. The pulse waveform parameters of the MBR that are synchronized with the heart rate and reported to be related to systemic vascular function include the blowout score (BOS), blowout time (BOT), and rising rate (RR) [12,13,14]. For example, associations between the BOT, BOS, and macrovascular arterial stiffness [12] left diastolic ventricular function [13], and the RR is associated with left ventricular systolic function [13]. Although the items obtained and evaluated when using LSFG are not assigned units, interindividual comparisons are possible when analyses are conducted using data from various basic and clinical studies [14,15,16].

We thus hypothesized that MetS and its components may lead to the onset of ocular diseases via the ocular blood flow. An earlier study by our research group examined ocular blood flow in individuals with sleep apnea syndrome who met the diagnostic criteria for MetS, in whom we observed decreases in the MBR in the optic nerve head (ONH) and choroid area and in the BOS in the choroidal region [17]. However, that study was conducted on a small number of older patients with sleep apnea syndrome (*n* = 76), with an average age in the 60s. An additional limitation of that study was that although we performed a multiple regression analysis, the numbers of male and female patients were not balanced.

Further research is therefore necessary to clarify the precise relationships between MetS and its components and ocular blood flow. We conducted this study to reinvestigate ocular blood flow in a large number of male patients with MetS in comparison with age-adjusted healthy subjects.

## 2. Materials and Methods

### 2.1. Subjects

Between December 2016 and December 2018, 853 males underwent ocular blood flow examination by LSFG during a physical examination at the JCHO Tokyo Kamata Medical Center. A total of 138 subjects satisfied the diagnostic criteria for MetS and were placed in a MetS group (49.95 ± 8.21 years). To reduce the number of background factors, we then conducted propensity score-matching with included subjects who did not exhibit abnormal glucose tolerance, hypertension, or dyslipidemia, using age as the covariate. A final total of 138 healthy males comprised the control group (49.96 ± 8.24 years). Subjects who had experienced a cardiovascular or cerebrovascular event, systemic disease such as arrhythmia, ocular disease (glaucoma, uveitis, optic neuropathy, and retinal diseases, etc.), or intraocular surgery were excluded due to the potential impact on their ocular blood flow.

### 2.2. Study Design

This was a retrospective analysis that used data obtained in our previous studies [9,10]. The study information was made available to the public, and the subjects had the opportunity to opt out of participating in the study. The study was approved by the Ethics Committee of Toho University Omori Hospital (approval no. M23044) and conducted according to the tenets of the Declaration of Helsinki. Although this study was a sub-analysis of the two studies reported by Kobayashi et al. in 2019 [9], after renewed submission to and approval by the Ethics Committee, we performed our analysis in the same patients, who were once again given the option to opt out if they so desired.

### 2.3. LSFG Measurements

**Figure 1:**
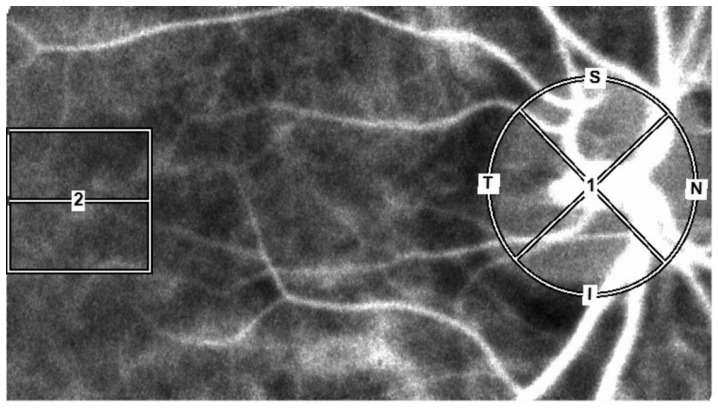
The LSFG measurement and waveform parameters. The LSFG measurement area settings (optic nerve head area and macula area) are shown.

LSFG measurements were performed as described in our previous study [9]. Briefly, an LSFG-NAVI™ instrument (Softcare Co., Fukuoka, Japan) was used to measure the subject’s MBR after an elliptical circle was set along the papillary margin for the ONH blood flow and a 150 × 150-pixel square was set in the macula for the choroidal blood flow. The use of this method avoided measurement of the retinal vessels (Figure 1).

**Figure 2:**
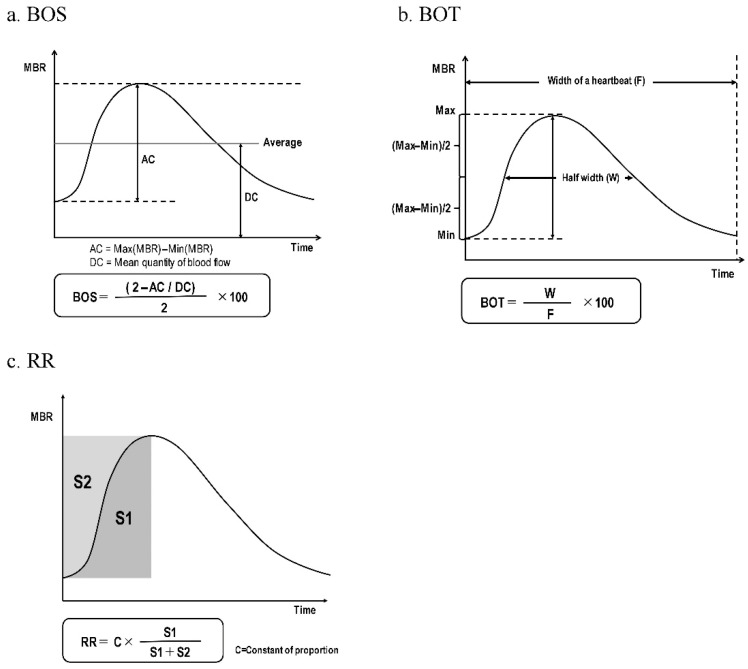
The waveform parameters calculated from the MBR waveforms. (**a**) Blowout score (BOS): This value indicates the blood flow rate for the amount of blood that flows through a blood vessel during one heartbeat and is based on the mean blood flow rate and the variation range of blood flow values. BOS = (2 − AC/2DC) × 100 (%). AC = MBR maximum − minimum (i.e., the MBR range of variation) DC = The average of the MBR values. (**b**) Blowout time (BOT): The percentage of time that is the half-width of the MBR that occupies one heartbeat. BOT = W/F × 100 (%). W: the time occupied by the MBR half-width, F: the time of one heartbeat. (**c**) Rising rate (RR): the area ratio of the area under curve in the ascending region of the MBR waveform.

For the ONH region, the MBR was calculated using two gradations, i.e., the vascular region (-vessel) and the tissue region (-tissue), for the entire ONH region (-all). We then averaged the data obtained from 4 sec of continuous ocular blood flow measurement by the LSFG instrument in order to calculate the MBR of the ONH region and the MBR of the choroidal region (-choroid) for one heartbeat. The BOS, BOT, and RR were calculated from the MBR using the LSFG instrument’s analytical software (ver. 3.2.3.0, Softcare Co.) (Figure 2a–c) [18].

All ophthalmic parameters were examined in the right eye of each subject.

### 2.4. Systemic, Laboratory, and Ophthalmic Parameter Measurements

The systemic parameters included age, body mass index (BMI, kg/m^2^), waist circumference (cm), systolic blood pressure (SBP, mmHg), diastolic blood pressure (DBP, mmHg), and heart rate (beats per minute, bpm), with clinical biochemistry and hematology parameters obtained from fasting morning blood draws. The ophthalmic parameters were collected from the LSFG data, along with the spherical refraction (diopter, D) and intraocular pressure (IOP mmHg), which were measured by non-contact tonometry. The subjects’ fasting blood sugar (FBS, mg/dL), triglycerides (TG, mg/dL), high-density lipoprotein cholesterol (HDL-C, mg/dL), low-density lipoprotein cholesterol (LDL-C, mg/dL), hematocrit (%) and glycated hemoglobin A1c (HbA1c %) were assessed in fasting morning blood samples.

### 2.5. Diagnosis of MetS

The diagnosis of MetS was based on the diagnostic criteria published by the Japanese Metabolic Syndrome Diagnostic Study Committee [19]. MetS is defined as abdominal obesity (waist ≥ 85 cm) with two or more of the following three criteria: (1) hypertension—SBP ≥ 130 mmHg or DBP ≥ 85 mmHg or a history of hypertension treatment; (2) dyslipidemia—HDL-C < 40 mg/dL or TG ≥ 150 mg/dL or a history of dyslipidemia treatment; and (3) glucose tolerance—FBS ≥ 110 mg/dL or a diagnosis of diabetes mellitus.

### 2.6. Statistical Analyses

We compared the above-mentioned parameters between the MetS and control groups using an unpaired *t*-test and the χ^2^-test. Single and multiple regression analyses were used to determine the relationships among the prevalence of MetS, its components, the MBR, and the parameters that showed a significant difference between the MetS and control groups. All statistical analyses were performed by EZR (Saitama Medical Center, Jichi Medical University, Saitama, Japan, ver. 1.54) [20]. Probability (*p*-) values < 0.05 were considered significant. Data for the continuous variables are presented as the mean ± standard deviation.

## 3. Results

After propensity score matching of the control groups using age as the covariate, 138 males were allocated to the MetS group, and 138 males comprised the control group (49.95 ± 8.21 vs. 49.96 ± 8.24 yrs, *p* = 0.99). Table 1 summarizes the subject backgrounds for each group.

Compared to the control group, the MetS group had significantly higher systemic or laboratory parameters (BMI, SBP, DBP, FBS, TG, hematocrit, and HbA1c) and significantly lower HDL-C values. Among the ocular parameters, a significantly higher IOP (*p* = 0.002) was observed in the MetS group compared to the control group.

Table 2 provides the results of a comparison of the groups’ MBR values. There was a significantly lower MBR-Choroid (*p* = 0.02) in the MetS subjects versus the control subjects. In the ONH region, there were no significant differences between the MetS and control groups for the MBR-all (*p* = 0.28), MBR-tissue (*p* = 0.19), and MBR-vessel (*p* = 0.85).

A comparison of LSFG waveform parameters between the MetS and control groups is summarized in Table 3. The BOS-All (*p* = 0.005), BOS-Tissue (*p* = 0.04), and BOS-Vessel (*p* = 0.002) were significantly higher in the MetS group versus the control group. The BOS-Choroid values in the MetS group tended to be higher than those of the control group (*p* = 0.07). The MetS group’s RR-all (*p* < 0.001), RR-tissue (*p* < 0.001), RR-vessel (*p* < 0.001), and RR-Choroid (*p* < 0.001) values were significantly lower than those of the control group. There was no significant difference in BOT results between the two groups.

We then conducted a single-regression analysis to investigate whether the number of MetS components contributes independently to the MBR-Choroid value (Table 4). Among the parameters evaluated, the number of MetS components was only significantly negatively correlated with the MBR-Choroid (r = −0.14, *p* = 0.02).

We then performed single- and multiple-regression analyses to determine which MetS component was most strongly correlated with the MBR-Choroid. The explanatory variables were correlated significantly with the objective variables in the single-regression analysis (Table 5). Because the correlation coefficients between HbA1c and FBS and between BMI and waist circumference were >0.8, we selected HbA1c and waist circumference as the explanatory variables that demonstrated stronger correlations with the objective variables. The analysis revealed that HbA1c was the only factor that contributed independently to the MBR-Choroid (β = −0.45, t-value = −2.25, *p* = 0.03).

## 4. Discussion

MetS has been recognized as an important risk factor for not only systemic arteriosclerotic and atherosclerotic diseases but also ocular diseases such as glaucoma and retinal vein occlusion [1,2,3,4,5]. It would thus be very informative to determine the effects of MetS on micro-hemodynamics before the onset of systemic or ocular disease. A previous study by our research group focused on the relationship between ocular hemodynamics and MetS, and we observed the ocular blood flow in sleep apnea syndrome patients who had MetS, finding decreases in the MBR in the ONH and choroid and in the BOS-Choroid [17]. There were several matters to resolve in that study, however. Although multiple-regression analysis was performed, the study was conducted in a small number of elderly subjects, and both genders were included. Since gender-related differences in ocular microcirculation have been revealed, further investigation is needed.

In the present study, we re-examined ocular blood flow by LSFG in a large number of male subjects with MetS and compared the results with healthy age-adjusted subjects. All enrolled subjects were selected from among individuals who underwent physical examinations. As a result, these subjects were >10 years younger than the participants in the earlier study [17], and there was also a difference in the duration of MetS compared to the earlier study (MetS group 49.95 ± 8.21 years).

In this study, our comparison of background factors between the MetS and control groups matched for age demonstrated that the heart rate, hematocrit, and IOP were significantly higher in the MetS group than in the control group. It has been reported that heart rate is associated with obesity and diabetes mellitus, both of which are MetS components [21], and a high heart rate is an independent long-term predictor of death [22,23]. Several descriptions of relationships between MetS and ocular findings are available, and it has been reported that subjects with a greater number of MetS components have higher IOP values [24]. Relationships between erythrocyte parameters and MetS and its components were described [25]. Together, these reports may support our present findings.

Our analysis of ocular blood flow in the present MetS and control groups revealed that although the MBR-Choroid was significantly lower in the MetS group, there was no significant difference in MBR values in the ‘all’ section of the ONH (Table 2). This may be because the subjects in this study were >10 years younger than the subjects in the previous reports, in addition to the difference in the duration of MetS compared to the earlier studies. In the next 10 years, our subjects’ MBR-tissue and MBR-all values could also change and should thus be examined in a continuous study. In other words, our findings indicate that the decreasing choroidal blood flow indicated by the MBR may be present from the early stage of MetS.

As summarized in Table 3, we compared the MetS and control groups’ values for the BOS, BOT, and RR, which are LSFG pulse waveform parameters. Although the BOS-Tissue and Vessel values were higher and the RR was lower in the MetS group, no significant differences were observed for the BOT. Our group has reported that the BOT and BOS in the ONH were significantly negatively correlated with carotid arterio-atherosclerotic formation [12], and heart rate was reported to be strongly positively correlated with the BOS [17]. This result may indicate that heart rate contributes more strongly to the BOS compared to atherosclerotic changes in the early stages of MetS. Regarding the RR, especially in the choroid area, our group has demonstrated that it reflects left ventricular function [13]. It was reported that MetS components may have a substantial effect on the development of cardiac heart failure [26]. It was also proposed that MetS poses a risk of preclinical heart failure [27]. Further investigations are therefore necessary to determine the exact relationships between cardiac hemodynamics obtained by echocardiography and ocular blood flow in individuals with MetS.

Table 4 provides the results of the single-regression analysis conducted to determine whether the accumulation of MetS components affects the MBR-Choroid. Among the background factors examined, only the number of MetS components showed a significant correlation and was significantly negatively correlated with the MBR-Choroid. This result reconfirms that the overlap of MetS components is an important factor in defining ocular blood flow in the choroid.

Finally, we conducted single- and multiple-regression analyses to determine the MetS-related factor (s) that most affect the MBR-Choroid (Table 5). The multiple-regression analysis identified HbA1c as a factor contributing independently to the MBR-Choroid. Our findings clarified that in the early stage of MetS, (i) MetS subjects had lower ONH and choroid RR values (which reflect left ventricular function), and (ii) the MBR in the choroid area decreases in parallel with the accumulation of MetS components. Our results also clarified that the MetS component with the strongest influence on the MBR-Choroid was the HbA1c value, which is related to blood sugar. Studies of patients with diabetes have reported that ocular blood flow is reduced even before the onset of diabetic retinopathy [28], and there is a gradual decrease in ocular blood flow in the ONH in conjunction with the progression of retinal vascular morphology changes [29,30].

In MetS, ocular blood flow is expected to decrease not only the choroidal blood flow but also the ONH blood flow if functional and morphological changes in the retinal vessels progress. In any case, our present findings demonstrate reduced choroidal blood flow, even under conditions considered to indicate a relatively early stage of MetS. These results contribute to the elucidation of the pathology of progressive systemic and ocular diseases related to MetS from the perspective of ocular microcirculation. Further prospective observational studies will be needed to determine whether managing MetS, e.g., through lifestyle modifications (diet and exercise), can improve ocular blood flow and prevent or slow the progress of eye complications.

This study has several major study limitations to consider. First, only males were examined because participants in our medical checkup program were more often men than women, resulting in a clear difference in the numbers of men and women in this study. Thus, our findings should not be generalized to females. Further studies in both genders with larger patient populations are required. The participants in this study were recruited from a medical checkup program; therefore, some important laboratory and physiological factors could not be investigated, meaning that hyperlipoproteinemia, homocysteinemia, endothelial dysfunction, and ocular motor cranial nerve palsy cannot be completely ruled out. In addition, because fundus photographs and optical coherence tomography data were not available for all subjects, retinal vessel changes, glaucoma risk, and retinal and choroidal diseases cannot be completely ruled out.

The study data were extracted from a large database to enable a matched pair analysis, which could have led to selection bias. Although fundus photographs were taken for all subjects, optical coherence tomography was not performed for all subjects, meaning that retinal and choroidal diseases cannot be completely ruled out. Finally, because the information on ocular and systemic diseases was obtained primarily from medical interviews of the subjects, there is a chance that other comorbidities or treatments for diseases such as hypertension and diabetes were overlooked. Due to the nature of the physical examinations, it is possible that the participants did not strictly adhere to restrictions on fasting, alcohol consumption, and smoking prior to the examination. Further careful validation studies are necessary to overcome these limitations.

## 5. Conclusions

Our study of males clarified that the participants with early-stage MetS had a lower rising rate (RR, which reflects the left ventricular function) and an MBR in the choroid area that was decreasing in parallel with the accumulation of the MetS components. The MetS component with the strongest influence on the MBR-Choroid was the HbA1c value, which is related to blood sugar.

## Figures and Tables

**Table 1:** Comparison of the MetS and control groups by background factors.

	MetS *n* = 138	Control *n* = 138	*p*-Value
Gender, male	–	–	
Age, yrs	49.95 ± 8.21	49.96 ± 8.24	0.99 *
BMI, kg/m^2^	27.85 ± 3.38	22.41 ± 2.53	<0.001 *
Waist circ., cm	95.24 ± 7.78	81.03 ± 7.50	<0.001 *
SBP, mmHg	137.36 ± 17.14	112.75 ± 10.31	<0.001 *
DBP, mmHg	87.88 ± 12.43	70.61 ± 7.22	<0.001 *
Pulse pressure, mmHg	49.49 ± 11.43	42.14 ± 7.03	<0.001 *
Heart rate, bpm	75.78 ± 10.49	67.98 ± 9.26	<0.001 *
FBS, mg/dL	121.43 ± 31.01	95.46 ± 7.80	<0.001 *
TG, mg/dL	212.91 ± 164.75	91.33 ± 28.89	<0.001 *
HDL-C, mg/dL	52.30 ± 13.38	64.98 ± 14.40	<0.001 *
LDL-C, mg/dL	139.09 ± 35.78	129.18 ± 27.14	0.010 *
Hematocrit, %	45.92 ± 3.17	44.22 ± 3.11	<0.001 *
HbA1c, %	6.18 ± 0.94	5.53 ± 0.26	<0.001 *
Spherical refraction, D	−2.35 ± 2.65	−2.07 ± 2.67	0.38 *
IOP, mmHg	12.61 ± 3.10	11.54 ± 2.69	0.002 *
Glucose tolerance, %	92 (66.7)	0 (0)	<0.001 **
Dyslipidemia, %	115 (83.3)	0 (0)	<0.001 **
Hypertension, %	123 (89.1)	0 (0)	<0.001 **

* Unpaired *t*-test, ** χ^2^-test. BMI: body mass index, bpm: beats per minute, circ: circumference, D: diopter, DBP: diastolic blood pressure, FBS: fasting blood sugar, HbA1c: glycated hemoglobin A1c, HDL-C: high-density lipoprotein cholesterol, IOP: intraocular pressure, LDL-C: low-density lipoprotein cholesterol, MetS: metabolic syndrome, SBP: systolic blood pressure, TG: triglyceride.

**Table 2:** Comparison of the MBR between the MetS and control groups.

MBR (AU)	MetS *n* = 138	Control *n* = 138	*p*-Value
MBR-All	24.64 ± 4.09	25.20 ± 4.36	0.28
MBR-Tissue	12.67 ± 2.49	13.07 ± 2.47	0.19
MBR-Vessel	44.84 ± 6.40	44.99 ± 7.06	0.85
MBR-Choroid	8.81 ± 2.82	9.59 ± 2.57	0.02

*p*-values: unpaired *t*-test. AU: arbitrary unit, MBR: mean blur rate.

**Table 3:** Comparison of LSFG waveform parameters between the MetS and control groups.

Parameter (AU)	MetS *n* = 138	Control *n* = 138	*p*-Value
BOS-All	81.72 ± 4.50	80.31 ± 3.80	0.005
BOS-Tissue	78.78 ± 4.95	77.64 ± 4.09	0.04
BOS-Vessel	83.02 ± 4.32	81.51 ± 3.79	0.002
BOS-Choroid	77.93 ± 5.36	76.80 ± 4.99	0.07
BOT-All	52.75 ± 4.66	53.18 ± 3.77	0.40
BOT-Tissue	49.92 ± 4.79	50.35 ± 3.64	0.41
BOT-Vessel	54.23 ± 4.74	54.70 ± 4.04	0.38
BOT-Choroid	48.67 ± 5.03	49.32 ± 3.53	0.21
RR-All	12.64 ± 0.93	13.37 ± 0.85	<0.001
RR-Tissue	12.26 ± 0.85	12.91 ± 0.84	<0.001
RR-Vessel	12.79 ± 1.01	13.62 ± 1.00	<0.001
RR-Choroid	12.24 ± 0.91	12.78 ± 0.95	<0.001

*p*-values: unpaired *t*-test. BOS: blowout score, BOT: blowout time, MetS: metabolic syndrome, RR: rising rate.

**Table 4:** Results of a single-regression analysis for factors contributing to the MBR-Choroid using the number of MetS components (*n* = 276).

Explanatory Variables	r	*p*-Value
Age, yrs	−0.054	0.37
Heart rate, bpm	0.049	0.42
Hematocrit, %	−0.089	0.14
Spherical refraction, D	−0.030	0.63
IOP, mmHg	0.094	0.12
MetS component, number	−0.14	0.02

Objective variable: MBR-Choroid. bpm: beats per minute; D; diopter; IOP: intraocular pressure; Mets: metabolic syndrome; yrs: years.

**Table 5:** Results of single- and multiple-regression analyses for factors independently contributing to MBR-Choroid (*n* = 276).

Explanatory Variables	Single Regression		Multiple Regression		
	r	*p*-Value	β	t-Value	*p*-Value
SBP, mmHg	−0.12	0.05			
DBP, mmHg	−0.12	0.05			
HbA1c, %	−0.22	0.001	−0.45	−2.25	0.03
FBS, mg/dL	−0.15	0.02			
TG, mg/dL	−0.14	0.02	−0.14	−0.69	0.49
LDL-C, mg/dL	−0.099	0.10			
HDL-C, mg/dL	0.14	0.02	0.31	1.54	0.13
BMI, kg/m^2^	−0.11	0.01			
Waist circ., cm	−0.15	0.01	−0.041	−0.20	0.84

Objective variable: MBR-Choroid. BMI: body mass index; circ.: circumference; DBP: diastolic blood pressure; FBS: fasting blood sugar; HbA1c: glycated hemoglobin A1c; HDL-C: high-density lipoprotein cholesterol; LDL-C: low-density lipoprotein cholesterol; MetS: metabolic syndrome; SBP: systolic blood pressure; TG: triglyceride.

## Data Availability

The datasets generated and/or analyzed during the current study are not publicly available due to data privacy concerns but are available from the corresponding author on reasonable request.

## Abbreviations

MetS	Metabolic syndrome
LSFG	Laser speckle flowgraphy
MBR	Mean blur rate
BOS	Blowout score
BOT	Blowout time
RR	Rising rate
ONH	Optic nerve head
BMI	Body mass index
SBP	Systolic blood pressure
DBP	Diastolic blood pressure
bpm	Beat per minute
D	Diopter
IOP	Intraocular pressure
FBS	Fasting blood sugar
TG	Triglycerides
HDL-C	High-density lipoprotein cholesterol
LDL-C	Low-density lipoprotein cholesterol
HbA1c	Glycated hemoglobin A1c
